# Synthesis and structure of 2-amino-4-methyl­pyridin-1-ium hippurate dihydrate

**DOI:** 10.1107/S2056989026004846

**Published:** 2026-05-22

**Authors:** Vanitha Vetrivel, Nishandhini Marimuthu, Thangavelu Balakrishnan

**Affiliations:** a Crystal Growth Laboratory PG and Research department of Physics Thanthai Periyar Government Arts and Science College (Autonomous) affiliated to Bharathidasan University, Tiruchirappalli Tiruchirappalli-620 023 Tamil Nadu India; b Department of Bioinformatics, VISTAS, Chennai, Tamil Nadu, India; University of Aberdeen, United Kingdom

**Keywords:** crystal structure, 2-amino-4-methyl­pyridin-1-ium cation, hippurate anion, Hirshfeld surface analysis

## Abstract

In the extended structure of the title salt, C_6_H_9_N_2_^+^·C_9_H_8_NO_3_^−^·2H_2_O, the 2-amino-4-methyl­pyridin-1-ium cation and hippurate [or 2-(phenyl­formamido)­acetate] anion are linked through paired N—H⋯O hydrogen bonds, forming an *R*_2_^2^(8) motif. The water mol­ecules of crystallization participate in O—H⋯O and N—H⋯O hydrogen-bonding inter­actions, which connect the mol­ecular components into one-dimensional chains that extend along the [010] direction. These inter­actions collectively generate a three-dimensional supra­molecular network.

## Abstract 2

In the title salt, the crystal structure features N—H⋯O and O—H⋯O hydrogen-bonding inter­actions, which link the components into infinite chains and generate an extended supra­molecular network.

## 1. Chemical context

Pyridinium-based organic salts continue to attract inter­est owing to their diverse supra­molecular architectures and their ability to form robust hydrogen-bonded networks in the solid state (Konovalova & Reiss, 2025 ▸; Bis & Zaworotko, 2005 ▸; Budzikur *et al.*, 2022 ▸). 2-Amino-4-methyl­pyridine, C_6_H_8_N_2_, is a bi-functional heterocycle containing both a basic pyridine nitro­gen (ring N atom) and an exocyclic amino group (-NH_2_), enabling different hydrogen-bonding patterns and facilitating salt formation with a variety of organic acids. Protonated amino­pyridines are widely used as structure-directing cations because their multiple donor and acceptor sites support extended N—H⋯O, O—H⋯O, and π-associated supra­molecular motifs in the solid-state (Bedeković *et al.*, 2017 ▸; Desiraju, 2002 ▸; Aakeröy & Seddon, 1993 ▸).

Hippuric acid (benzoyl­glycine, C_9_H_9_NO_3_) is a biologically relevant carb­oxy­lic acid that mimics short peptide fragments and provides several potential donor/acceptor sites through its carboxyl, amide, and aromatic groups (Li *et al.*, 2024 ▸). Both hippuric acid and its deprotonated hippurate (benzoyl­glycinate, C_9_H_8_NO_3_^−^) anions are widely employed in organic salts and co-crystals, where their amide, carboxyl­ate, and aromatic functionalities enable complementary hydrogen-bonding inter­actions and π-stacking contacts (Laishram *et al.*, 2025 ▸; Suganya *et al.*, 2021 ▸).

Proton transfer in crystalline acid–base systems is commonly rationalized using the Δp*K*_a_ rule (Cruz-Cabeza, 2012 ▸, 2022 ▸): when the difference between the p*K*_a_ of the conjugate acid of the base and the p*K*_a_ of the acid exceeds ≃ 2–3, salt formation is favored. For the present system, the reported p*K*_a_ values (hippuric acid ≃ 3.6 and 2-amino-4-methyl­pyridinium ion ≃ 7.5–8.1) gives Δp*K*_a_ ≃ 3.9, supporting proton transfer and the formation of a 2-amino-4-methyl­pyridinium benzoyl­glycinate salt. Such a proton transfer leads to the formation of charge-assisted N^+^—H⋯O hydrogen bonds, which are typically shorter, and more electrostatically strengthened than their neutral counterparts; these inter­actions often dominate the mol­ecular packing, enhance crystal cohesion and facilitate the incorporation of water mol­ecules of crystallization that further connect the ions through O—H⋯O and O—H⋯N hydrogen bonding.
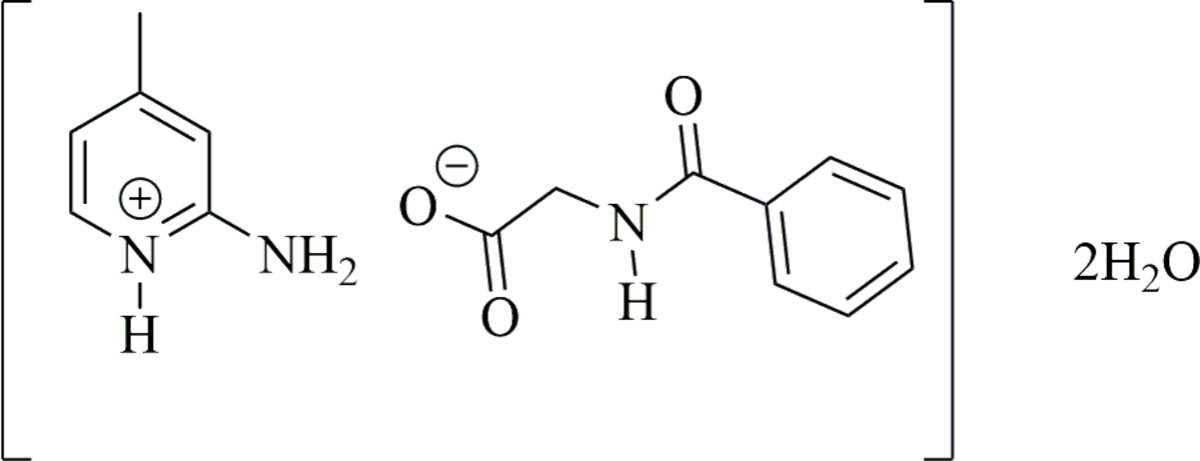


As part of our studies in this area, we now describe the synthesis, structure and Hirshfeld surface analysis of the title hydrated salt, C_6_H_9_N_2_^+^·C_9_H_8_NO_3_^−^·2H_2_O (**I**).

## 2. Structural commentary

The hydrated title salt (**I**) was obtained by proton transfer from hippuric acid to 2-amino-4-methyl­pyridine in aqueous solution. The crystal structure unambiguously confirms salt formation through a proton-transfer reaction, which is consistent with the acidity constants of the components noted above, which strongly favors salt formation rather than co-crystallization

**Figure 1:**
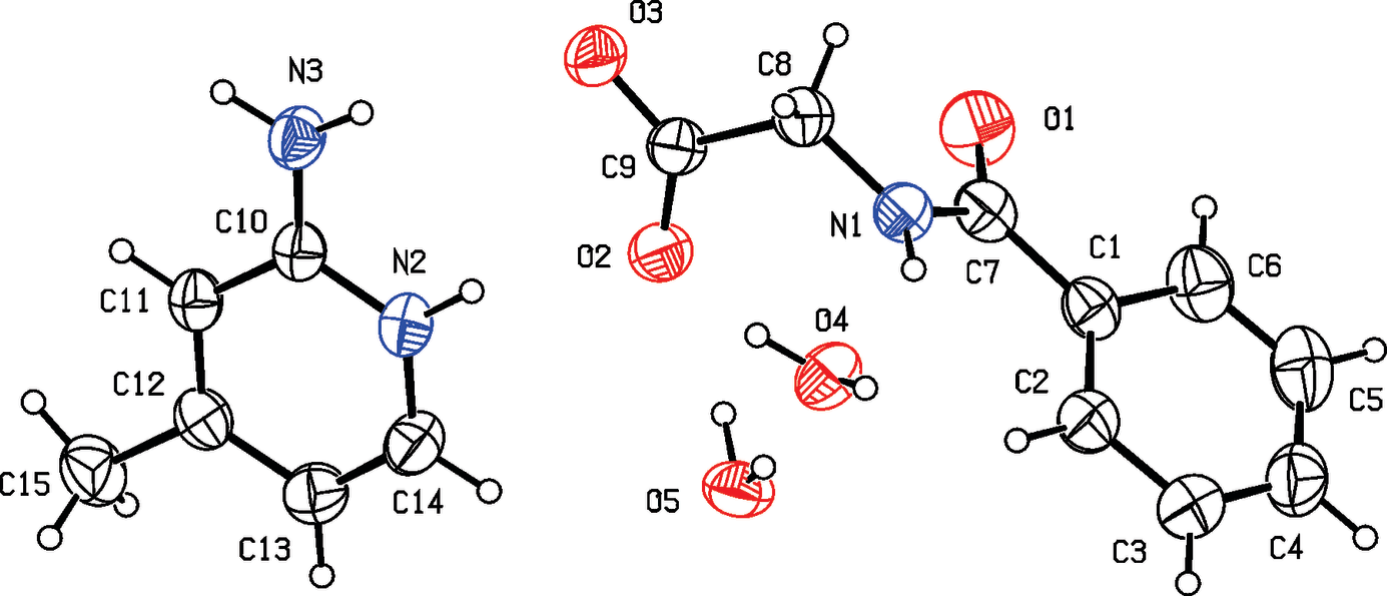
The mol­ecular structure of the title salt, (**I**), showing the atom-labelling scheme. Displacement ellipsoids are drawn at the 50% probability level.

Compound (**I**) crystallizes as ortho­rhom­bic in space group *Pbca*. The asymmetric unit consists of one 2-amino-4-methyl­pyridin-1-ium cation, one hippurate anion and two water mol­ecules of crystallization, as illustrated in Fig. 1 ▸. In the cation, proton migration to the pyridine nitro­gen atom (N2) is further supported by the increase in the inter­nal angle around the protonated nitro­gen atom [C10—N2—C14 = 122.22 (13)°], compared with 117.3 (1)° in neutral 2-amino-4-methyl­pyridine (Kvick & Noordik, 1977 ▸). The bond lengths and angles of the cation closely resemble those observed in related structures, including 2-amino-4-methyl­pyridin-1-ium hydrogen squarate (Vetrivel *et al.*, 2025 ▸) and other similar protonated analogues (Khalib *et al.*, 2014 ▸). The non-hydrogen atoms of the cation are essentially planar, with a maximum deviation of 0.027 (3) Å for atom C15. In the hippurate anion, the carboxyl­ate group has nearly equivalent C—O bond lengths [O2—C9 = 1.2377 (18) Å and O3—C9 = 1.2598 (19) Å; Δ = 0.0221 Å], confirming deprotonation (Table 1 ▸). The key torsion angles for the side chain are C1—C7—N1—C8 = −175.96 (12)° and C7—N1—C8—C9 = −87.11 (18)° and the dihedral angle between the C1–C6 phenyl ring and the carboxyl­ate plane (O1/O2/C8/C9) is 70.96 (7)°. These geometric parameters are comparable to those reported for deprotonated hippurate anions in related crystal structures, such as cytosinium *N*-benzoyl­glycinate monohydrate (Görbitz & Sagstuen, 2004 ▸).

## 3. Supra­molecular features

**Figure 2:**
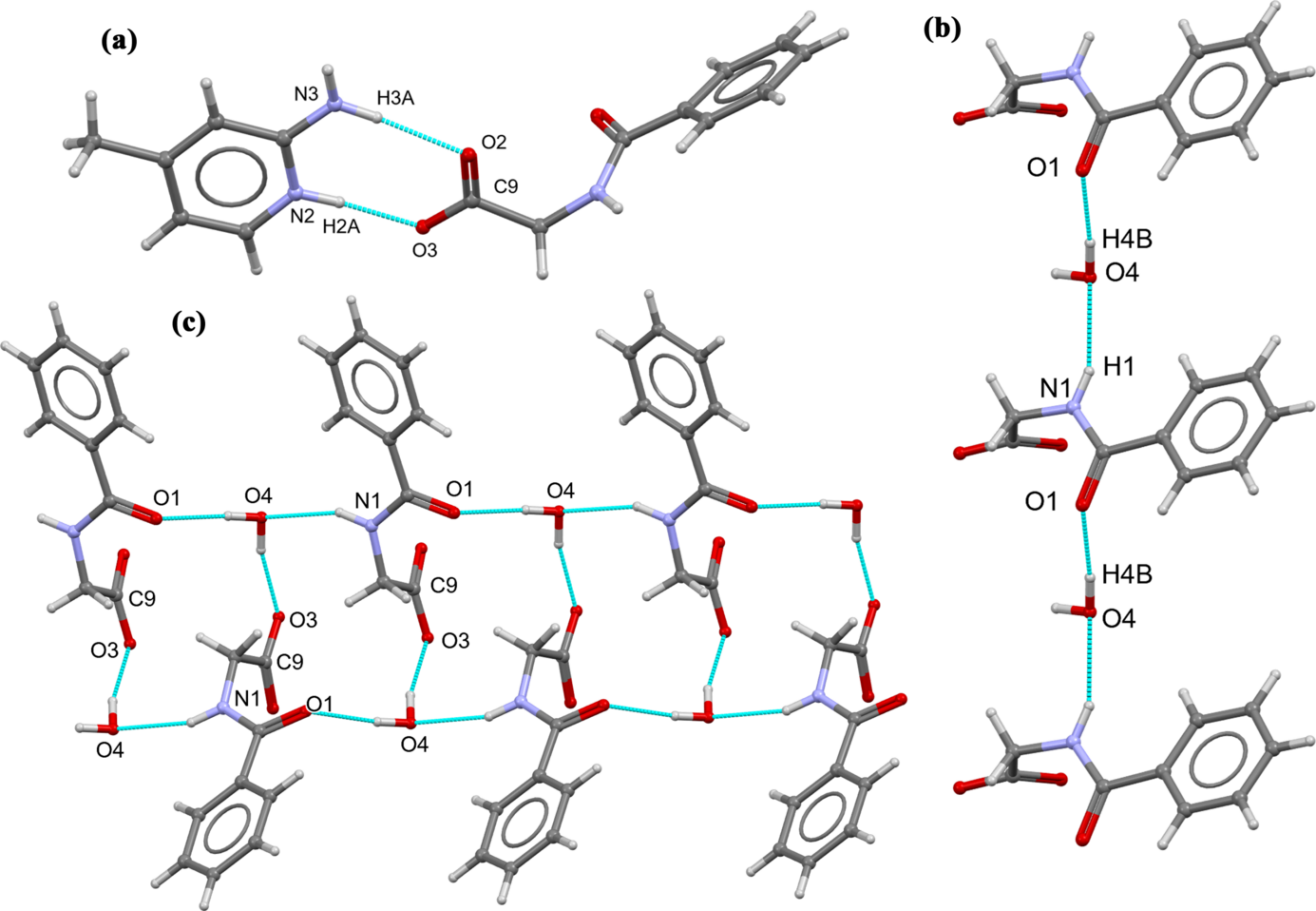
(*a*) Part of the crystal structure of (**I**) showing the 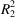
(8) motif formed by inter­molecular N—H⋯O hydrogen bonds. (*b*) The N1—H1⋯O4 and O4—H4*B*⋯O1 hydrogen bonds connect neighbouring hippurate anions and water mol­ecule O4 (water – 1), forming a one-dimensional chains runs along the [010] direction. (*c*) The O4—H4*A*⋯O3, N1—H1⋯O4 and O4—H4*B*⋯O1 hydrogen bonds generate an 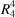 (16) motif, resulting in a supra­molecular ladder-like arrangement running parallel to [010].

In the extended structure, the cation and anion are connected through N2—H2*A*⋯O3 and N3—H3*A*⋯O2 hydrogen bonds (Table 1 ▸, Fig. 2 ▸), generating an 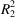 (8) motif. The two water mol­ecules of crystallization participate actively in the hydrogen-bonding network. In particular, a O5—H5*A*⋯O4 hydrogen bond links the two water mol­ecules. All the oxygen atoms of the anion (O1–O3), together with the water O atoms (O4 and O5), function as hydrogen-bond acceptors in various inter­molecular N—H⋯O and O—H⋯O inter­actions (Table 1 ▸).

**Figure 3:**
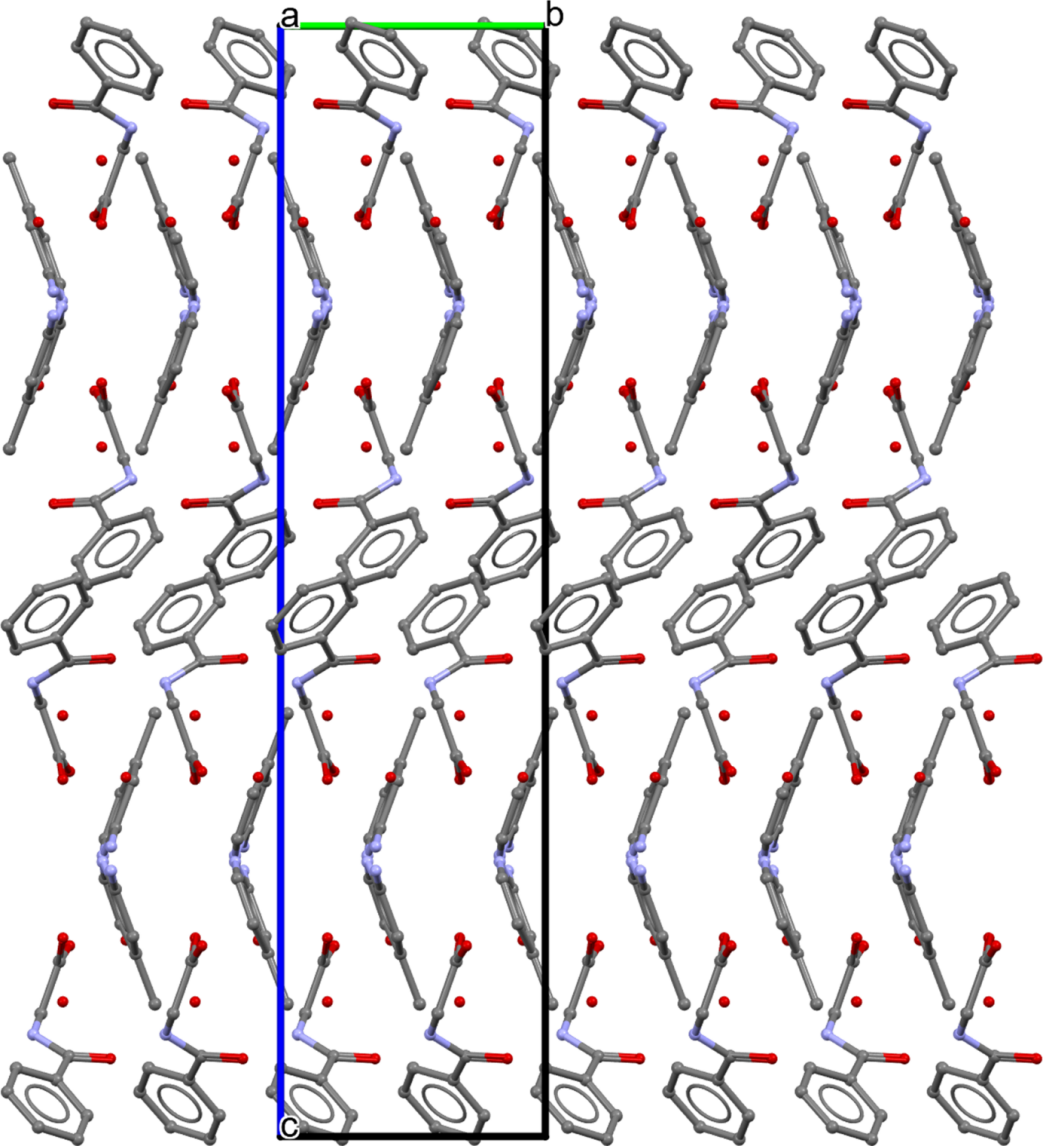
Overall crystal packing of the title salt (**I**), viewed down the *a* axis. Hydrogen atoms have been omitted for clarity.

The N1—H1⋯O4 and O4—H4*B*⋯O1 hydrogen bonds connect neighbouring hippurate anions and water mol­ecule (water-1, O4), forming four-membered units that propagate into a one dimensional chains extending along the [010] direction. Furthermore, the O4—H4*A*⋯O3 hydrogen bond along with the N1—H1⋯O4 and O4—H*4B*⋯O1 hydrogen bonds, generates an 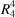 (16) loop, resulting in a supra­molecular ladder-like arrangement running parallel to [010]. This ladder is further reinforced by hydrogen bonding involving the cation, namely N2—H2*A*⋯O3, N3—H3*A*⋯O2 and O5—H5*B*⋯O2 and N3—H3*B·*··O5, which link the second water mol­ecule (water-2, O5) to the cationic fragment. Collectively, the hydrogen bonds inter­connect the cations, anions, and water mol­ecules of crystallization into a three-dimensional supra­molecular network (Figs. 3 ▸ and 4 ▸).

## 4. Hirshfeld surface analysis

**Figure 4:**
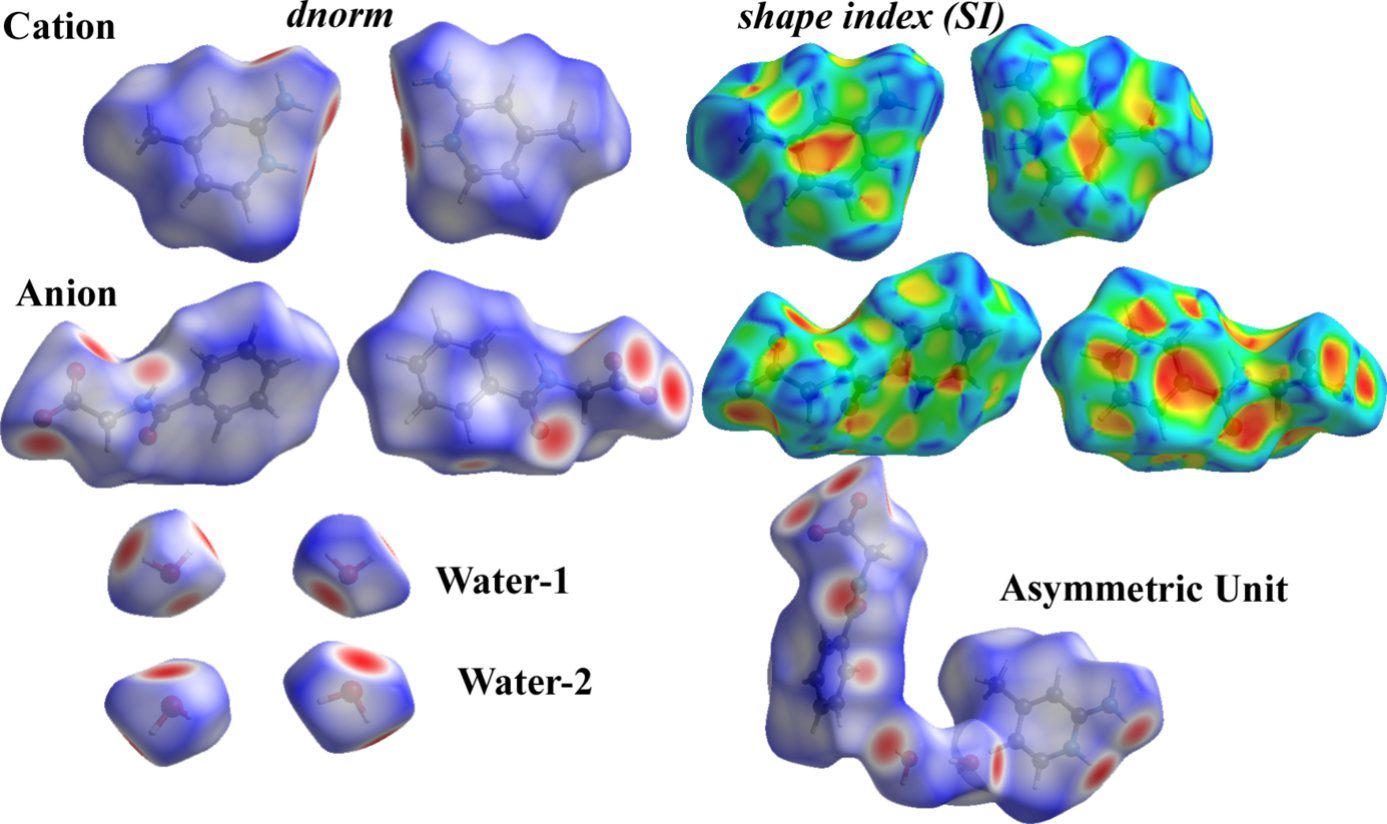
Two views of the Hirshfeld surfaces of the cation, anion and water molecules of crystallization in the title salt (**I**), mapped over *d*_norm_ and the shape-index surface.

Hirshfeld surface (HS) analysis was carried out using *CrystalExplorer 21.5* (Turner *et al.*, 2017 ▸). The front and back views of the HS mapped over *d*_norm_ for the asymmetric unit are shown in Fig. 4 ▸, together with the individual surfaces for the cation, anion and the two water mol­ecules. Bright-red spots on the *d*_norm_*-*mapped surfaces correspond to close contacts, *i.e*., inter­molecular distances shorter than the sum of the van der Waals radii, and thus indicate significant non-covalent inter­actions. In contrast, the shape-index surface does not exhibit complementary red and blue triangular features, indicating the absence of significant π–π stacking inter­actions in (**I**).

**Figure 5:**
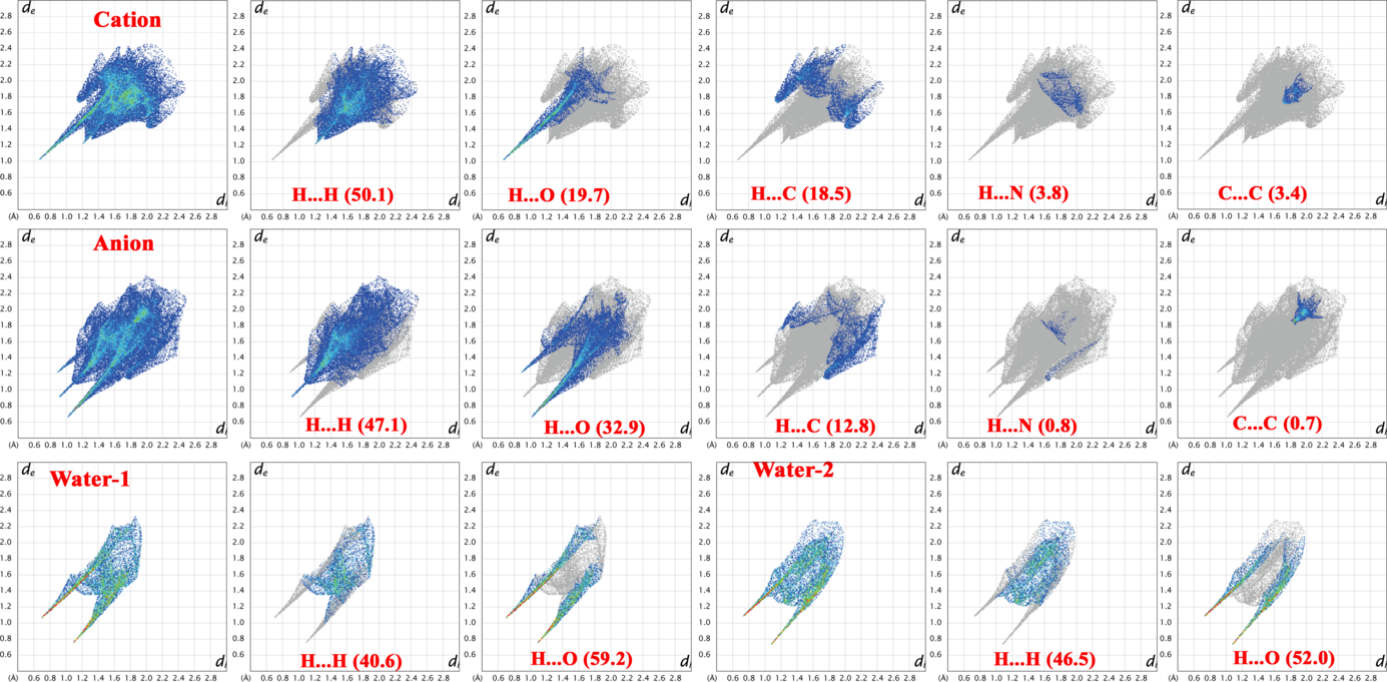
Full and decomposed two-dimensional fingerprint (FP) plots for the cation, anion and the two water molecules of crystallization in the title salt (**I**), showing the different inter­molecular contacts and their percentage contributions.

The full and decomposed two-dimensional fingerprint plots for the cation, anion and the water mol­ecules are presented in Fig. 5 ▸. The H⋯H contacts make the largest contribution for both the cation (50.1%) and the anion (47.1%), and also account for significant contributions in water-1 (40.6%) and water-2 (46.5%). For the water mol­ecules, O⋯H/H⋯O contacts are particularly prominent, reflecting their active participation in O—H⋯O hydrogen bonds. The sharp spikes observed in the FP plots at *d_e_* + *d*_i_ = 1.6–1.8 Å for O⋯H/H⋯O contacts are characteristic of strong N/O—H⋯O hydrogen bonds (Table 1 ▸). The C⋯H/H⋯C inter­actions represent the next significant contribution in the cation (18.5%) and anion (12.8%). The remaining contacts, namely N⋯H/H⋯N, C⋯N and C⋯C, contribute comparatively less to the total Hirshfeld surface area. Although both water mol­ecules participate in O—H⋯O hydrogen bonding, their relative percentage contributions and hydrogen-bond geometries indicate subtle differences in their inter­action environments within the crystal.

## 5. Database survey

A search of the Cambridge Structural Database (CSD, Version 6.01, updated of November 2025; (Groom *et al.*, 2016 ▸) performed using Conquest (Bruno *et al.*, 2002 ▸) for the 2-amino-4-methyl­pyridin-1-ium cation yielded 62 entries corresponding to salt forms. A number of these salts are with substituted benzoic acids such as 2-hy­droxy­benzoic acid (CSD refcode DUTZOI) and 3-hy­droxy­benzoic acid (AGAQIK) (Khalib *et al.*, 2013 ▸), 2- and 4-chloro­benzoic acids (COZVAQ and COZVOE); 4-methyl­benzoic acid (COZVIY) (Khalib *et al.*, 2014 ▸); and 4-nitro­benzoic acid (DUNCOF; Hemamalini & Fun, 2010*a* ▸), as well as other related substituted benzoates.

Salts with aliphatic carb­oxy­lic acids have also been reported, including succinic acid (DICYEW; Seth *et al.*, 2018 ▸), fumaric acid (DUSPUD; Hemamalini & Fun, 2010*c* ▸), tri­fluoro­acetic acid (KUSVAW; Hemamalini & Fun, 2010*b* ▸), sorbic acid (SUZXUH; Hemamalini & Fun, 2010*d* ▸), oxalic acid (YIZDAQ; Hemalatha *et al.*, 2023 ▸) and tartaric acid (YOHHIO; Jovita *et al.*, 2014 ▸).

A separate search for the hippurate anion revealed eight structures in the CSD. These correspond to salts of hippuric acid with various active pharmaceutical ingredients (APIs) and biologically relevant bases, including imatinib (AJIPOC) (Jiang *et al.*, 2025 ▸); ciprofloxacin (OSUQEA; Chadha *et al.*, 2016 ▸); cytosine (CYTBGL01); Görbitz & Sagstuen, 2004 ▸) guanidine (BEMWOJ; Reena *et al.*, 2022 ▸) and acridine (XANSOY; Suganya *et al.*, 2021 ▸). These results indicate that, although salts of the individual components are well documented in the structural database, structures comprising both components in a single salt are comparatively uncommon. The available entries further underscore the conformational flexibility and supra­molecular versatility of the hippurate anion, which consistently assembles into stable crystal architectures primarily via classical N—H⋯O and O—H⋯O hydrogen-bonding inter­actions.

## 6. Synthesis and crystallization

Hot methanol solutions (50 mL) of 2-amino-4-methyl­pyridine (1.08 g, 1.00 mmol) and hippuric acid (1.80 g, 1.00 mmol) were mixed and warmed over a heating magnetic stirrer hotplate for 6h. The reaction mixture was stirred at room temperature for 6 h to obtain a clear homogeneous solution. The resulting solution was filtered and allowed to evaporate slowly at room temperature. Colourless block-shaped crystals suitable for single-crystal X-ray diffraction were obtained after approximately 10 days.

## 7. Refinement

Crystal data, data collection and structure refinement details are summarized in Table 2 ▸. H atoms were positioned geometrically (C—H = 0.93–0.96 Å) and refined as riding with *U*_iso_(H) = 1.2*U*_eq_(C).

## Supplementary Material

Crystal structure: contains datablock(s) I. DOI: 10.1107/S2056989026004846/hb8212sup1.cif

Structure factors: contains datablock(s) I. DOI: 10.1107/S2056989026004846/hb8212Isup2.hkl

Supporting information file. DOI: 10.1107/S2056989026004846/hb8212Isup3.cml

CCDC reference: 2552915

Additional supporting information:  crystallographic information; 3D view; checkCIF report

## Figures and Tables

**Table 1:** Hydrogen-bond geometry (Å, °)

*D*—H⋯*A*	*D*—H	H⋯*A*	*D*⋯*A*	*D*—H⋯*A*
O5—H5*A*⋯O4	0.83 (2)	2.00 (2)	2.8225 (18)	167 (3)
N1—H1⋯O4^i^	0.85 (1)	2.25 (2)	2.9992 (18)	148 (2)
N2—H2*A*⋯O3^ii^	0.90 (2)	1.78 (2)	2.6850 (17)	176 (2)
N3—H3*A*⋯O2^ii^	0.89 (2)	1.99 (2)	2.8751 (17)	175 (2)
N3—H3*B*⋯O5^iii^	0.90 (2)	1.94 (2)	2.8368 (18)	171 (2)
O4—H4*A*⋯O3^iv^	0.88 (2)	1.88 (2)	2.7392 (17)	166 (2)
O4—H4*B*⋯O1^v^	0.87 (2)	1.95 (2)	2.8173 (18)	173 (2)
O5—H5*B*⋯O2^i^	0.88 (2)	1.94 (2)	2.8061 (18)	170 (3)

Symmetry codes: (i) 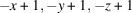; (ii) 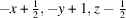; (iii) 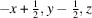; (iv) 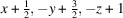; (v) 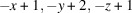.

**Table 2:** Experimental details

Crystal data	
Chemical formula	C_6_H_9_N_2_^+^·C_9_H_8_NO_3_^−^·2(H_2_O)
*M*_r_	323.35
Crystal system, space group	Orthorhombic, *Pbca*
Temperature (K)	298
*a*, *b*, *c* (Å)	15.137 (3), 7.3028 (14), 30.583 (6)
*V* (Å^3^)	3380.7 (11)
*Z*	8
Radiation type	Cu *K*α
μ (mm^−1^)	0.81
Crystal size (mm)	0.35 × 0.24 × 0.22

Data collection	
Diffractometer	Bruker D8 Venture Diffractometer
Absorption correction	Multi-scan (*SADABS*; Krause *et al.*, 2015 ▸)
*T*_min_, *T*_max_	0.547, 0.753
No. of measured, independent and observed [*I* > 2σ(*I*)] reflections	24560, 3208, 2918
*R*_int_	0.051
(sin θ/λ)_max_ (Å^−1^)	0.610

Refinement	
*R*[*F*^2^ > 2σ(*F*^2^)], *wR*(*F*^2^), *S*	0.053, 0.153, 1.09
No. of reflections	3208
No. of parameters	234
No. of restraints	8
H-atom treatment	H atoms treated by a mixture of independent and constrained refinement
Δρ_max_, Δρ_min_ (e Å^−3^)	0.20, −0.20

Computer programs: *APEX4*, *SAINT* and *XPREP* (Bruker, 2021 ▸), *SHELXT2014/5* (Sheldrick, 2015*a* ▸), *SHELXL2014/3* (Sheldrick, 2015*b* ▸), *ORTEP-3 for Windows* (Farrugia, 2012 ▸), *Mercury* (Macrae *et al.*, 2020 ▸) and *PLATON* (Spek, 2020 ▸).

## supplementary crystallographic information

### 2-Amino-4-methylpyridin-1-ium 2-(phenylformamido)acetate dihydrate Crystal data

**Table:**

C_6_H_9_N_2_^+^·C_9_H_8_NO_3_^−^·2(H_2_O)	*D*_x_ = 1.271 Mg m^−^^3^
*M**_r_* = 323.35	Cu *K*α radiation, λ = 1.54178 Å
Orthorhombic, *P**b**c**a*	Cell parameters from 7667 reflections
*a* = 15.137 (3) Å	θ = 4.1–70.0°
*b* = 7.3028 (14) Å	µ = 0.81 mm^−^^1^
*c* = 30.583 (6) Å	*T* = 298 K
*V* = 3380.7 (11) Å^3^	Block, colourless
*Z* = 8	0.35 × 0.24 × 0.22 mm
*F*(000) = 1376	

### 2-Amino-4-methylpyridin-1-ium 2-(phenylformamido)acetate dihydrate Data collection

**Table:**

Bruker D8 Venture Diffractometer	2918 reflections with *I* > 2σ(*I*)
Radiation source: micro focus sealed tube	*R*_int_ = 0.051
φ and ω scans	θ_max_ = 70.0°, θ_min_ = 2.9°
Absorption correction: multi-scan (SADABS; Krause *et al.*, 2015)	*h* = −18→18
*T*_min_ = 0.547, *T*_max_ = 0.753	*k* = −8→8
24560 measured reflections	*l* = −34→37
3208 independent reflections	

### 2-Amino-4-methylpyridin-1-ium 2-(phenylformamido)acetate dihydrate Refinement

**Table:**

Refinement on *F*^2^	Hydrogen site location: mixed
Least-squares matrix: full	H atoms treated by a mixture of independent and constrained refinement
*R*[*F*^2^ > 2σ(*F*^2^)] = 0.053	*w* = 1/[σ^2^(*F*_o_^2^) + (0.0923*P*)^2^ + 0.350*P*] where *P* = (*F*_o_^2^ + 2*F*_c_^2^)/3
*wR*(*F*^2^) = 0.153	(Δ/σ)_max_ = 0.001
*S* = 1.09	Δρ_max_ = 0.20 e Å^−^^3^
3208 reflections	Δρ_min_ = −0.19 e Å^−^^3^
234 parameters	Extinction correction: SHELXL2019/2 (Sheldrick 2015b), Fc^*^=kFc[1+0.001xFc^2^λ^3^/sin(2θ)]^-1/4^
8 restraints	Extinction coefficient: 0.0072 (6)

### 2-Amino-4-methylpyridin-1-ium 2-(phenylformamido)acetate dihydrate Special details

**Table:**

Geometry. All esds (except the esd in the dihedral angle between two l.s. planes) are estimated using the full covariance matrix. The cell esds are taken into account individually in the estimation of esds in distances, angles and torsion angles; correlations between esds in cell parameters are only used when they are defined by crystal symmetry. An approximate (isotropic) treatment of cell esds is used for estimating esds involving l.s. planes.

### 2-Amino-4-methylpyridin-1-ium 2-(phenylformamido)acetate dihydrate Fractional atomic coordinates and isotropic or equivalent isotropic displacement parameters (Å^2^)

**Table:**

	*x*	*y*	*z*	*U*_iso_*/*U*_eq_	
C1	0.52405 (11)	0.6531 (2)	0.55085 (5)	0.0634 (4)	
C2	0.57345 (12)	0.5010 (3)	0.56117 (6)	0.0820 (5)	
H2	0.553559	0.420177	0.582510	0.098*	
C3	0.65265 (14)	0.4676 (3)	0.53993 (7)	0.0976 (6)	
H3	0.685875	0.364962	0.547277	0.117*	
C4	0.68232 (13)	0.5843 (4)	0.50824 (6)	0.0949 (6)	
H4	0.735675	0.561428	0.494186	0.114*	
C5	0.63421 (17)	0.7325 (4)	0.49740 (7)	0.0992 (7)	
H5	0.654065	0.811233	0.475605	0.119*	
C6	0.55571 (15)	0.7679 (3)	0.51848 (7)	0.0886 (5)	
H6	0.523277	0.871148	0.510788	0.106*	
C7	0.43806 (12)	0.6994 (2)	0.57182 (5)	0.0653 (4)	
C8	0.30803 (10)	0.5937 (2)	0.61047 (5)	0.0666 (4)	
H8A	0.275980	0.478744	0.609411	0.080*	
H8B	0.276331	0.680558	0.592319	0.080*	
C9	0.30683 (10)	0.6635 (2)	0.65749 (5)	0.0614 (4)	
C10	0.22844 (9)	0.12490 (18)	0.27751 (4)	0.0547 (3)	
C11	0.24565 (10)	0.05872 (18)	0.32012 (4)	0.0588 (4)	
H11	0.198861	0.022428	0.337826	0.071*	
C12	0.32969 (10)	0.0475 (2)	0.33553 (5)	0.0636 (4)	
C13	0.40030 (11)	0.1037 (3)	0.30865 (6)	0.0742 (4)	
H13	0.458206	0.099824	0.318772	0.089*	
C14	0.38185 (11)	0.1635 (3)	0.26776 (6)	0.0743 (5)	
H14	0.427978	0.199248	0.249575	0.089*	
C15	0.34820 (14)	−0.0249 (3)	0.38085 (6)	0.0892 (6)	
H15A	0.365066	0.074466	0.399619	0.134*	
H15B	0.395280	−0.112843	0.379533	0.134*	
H15C	0.296011	−0.082495	0.392225	0.134*	
N1	0.39430 (9)	0.56514 (17)	0.59169 (4)	0.0621 (3)	
H1	0.4169 (11)	0.458 (2)	0.5917 (6)	0.074*	
N2	0.29813 (8)	0.17252 (17)	0.25277 (4)	0.0623 (3)	
H2A	0.2858 (11)	0.212 (2)	0.2254 (5)	0.075*	
N3	0.14821 (9)	0.1412 (2)	0.26100 (4)	0.0677 (4)	
O1	0.40736 (11)	0.85655 (16)	0.56980 (5)	0.0929 (4)	
O2	0.37639 (8)	0.67686 (18)	0.67853 (4)	0.0767 (4)	
O3	0.23079 (7)	0.70195 (18)	0.67150 (4)	0.0753 (4)	
H3A	0.1393 (13)	0.192 (3)	0.2350 (5)	0.090*	
H3B	0.1007 (11)	0.123 (3)	0.2784 (6)	0.090*	
O4	0.58180 (8)	0.82361 (16)	0.37897 (4)	0.0752 (4)	
H4A	0.6302 (14)	0.835 (3)	0.3632 (7)	0.113*	
H4B	0.5816 (16)	0.926 (3)	0.3936 (8)	0.113*	
O5	0.48776 (8)	0.5845 (2)	0.32339 (4)	0.0810 (4)	
H5A	0.5105 (18)	0.668 (3)	0.3383 (9)	0.122*	
H5B	0.5277 (16)	0.498 (3)	0.3257 (9)	0.122*	

### 2-Amino-4-methylpyridin-1-ium 2-(phenylformamido)acetate dihydrate Atomic displacement parameters (Å^2^)

**Table:**

	*U*^11^	*U*^22^	*U*^33^	*U*^12^	*U*^13^	*U*^23^
C1	0.0790 (9)	0.0570 (8)	0.0541 (7)	−0.0026 (6)	−0.0060 (6)	−0.0066 (6)
C2	0.0823 (11)	0.0888 (12)	0.0747 (10)	0.0142 (9)	0.0078 (8)	0.0155 (9)
C3	0.0814 (11)	0.1203 (17)	0.0911 (13)	0.0221 (11)	0.0085 (10)	0.0122 (12)
C4	0.0799 (11)	0.1351 (19)	0.0696 (10)	−0.0122 (12)	0.0047 (8)	−0.0119 (11)
C5	0.1157 (16)	0.1059 (16)	0.0761 (11)	−0.0271 (14)	0.0145 (11)	0.0030 (11)
C6	0.1159 (15)	0.0685 (10)	0.0816 (11)	−0.0032 (10)	0.0058 (10)	0.0065 (9)
C7	0.0876 (10)	0.0498 (8)	0.0586 (8)	0.0082 (7)	−0.0076 (7)	−0.0093 (6)
C8	0.0699 (9)	0.0698 (9)	0.0602 (8)	0.0084 (7)	−0.0062 (6)	−0.0154 (7)
C9	0.0645 (8)	0.0597 (8)	0.0600 (8)	0.0067 (6)	−0.0044 (6)	−0.0096 (6)
C10	0.0623 (8)	0.0475 (7)	0.0544 (7)	0.0030 (5)	0.0057 (5)	−0.0001 (5)
C11	0.0690 (8)	0.0530 (7)	0.0544 (7)	0.0020 (6)	0.0067 (6)	0.0014 (5)
C12	0.0738 (9)	0.0575 (8)	0.0595 (8)	0.0052 (6)	−0.0026 (6)	−0.0039 (6)
C13	0.0642 (8)	0.0820 (11)	0.0765 (10)	0.0032 (7)	−0.0022 (7)	0.0011 (8)
C14	0.0621 (8)	0.0838 (11)	0.0770 (10)	−0.0003 (7)	0.0113 (7)	0.0041 (8)
C15	0.0932 (12)	0.1075 (14)	0.0669 (10)	0.0085 (11)	−0.0128 (9)	0.0097 (9)
N1	0.0744 (8)	0.0522 (6)	0.0596 (7)	0.0106 (5)	−0.0007 (5)	−0.0081 (5)
N2	0.0652 (7)	0.0642 (7)	0.0574 (7)	0.0041 (5)	0.0092 (5)	0.0053 (5)
N3	0.0623 (7)	0.0802 (9)	0.0607 (7)	0.0035 (6)	0.0047 (5)	0.0136 (6)
O1	0.1266 (11)	0.0521 (7)	0.1000 (9)	0.0204 (6)	0.0054 (8)	−0.0046 (6)
O2	0.0679 (7)	0.0963 (8)	0.0658 (6)	0.0137 (5)	−0.0098 (5)	−0.0226 (5)
O3	0.0647 (7)	0.0951 (8)	0.0660 (6)	0.0101 (5)	−0.0031 (5)	−0.0206 (6)
O4	0.0689 (7)	0.0668 (7)	0.0900 (8)	−0.0026 (5)	0.0090 (6)	−0.0152 (6)
O5	0.0726 (7)	0.0918 (9)	0.0787 (8)	0.0119 (6)	−0.0210 (6)	−0.0044 (6)

### 2-Amino-4-methylpyridin-1-ium 2-(phenylformamido)acetate dihydrate Geometric parameters (Å, º)

**Table:**

C1—C2	1.376 (2)	C10—N2	1.3439 (18)
C1—C6	1.383 (3)	C10—C11	1.4142 (19)
C1—C7	1.490 (2)	C11—C12	1.359 (2)
C2—C3	1.385 (3)	C11—H11	0.9300
C2—H2	0.9300	C12—C13	1.409 (2)
C3—C4	1.367 (3)	C12—C15	1.510 (2)
C3—H3	0.9300	C13—C14	1.354 (3)
C4—C5	1.346 (3)	C13—H13	0.9300
C4—H4	0.9300	C14—N2	1.349 (2)
C5—C6	1.376 (3)	C14—H14	0.9300
C5—H5	0.9300	C15—H15A	0.9600
C6—H6	0.9300	C15—H15B	0.9600
C7—O1	1.2394 (19)	C15—H15C	0.9600
C7—N1	1.330 (2)	N1—H1	0.851 (14)
C8—N1	1.442 (2)	N2—H2A	0.904 (15)
C8—C9	1.526 (2)	N3—H3A	0.888 (15)
C8—H8A	0.9700	N3—H3B	0.903 (15)
C8—H8B	0.9700	O4—H4A	0.881 (17)
C9—O2	1.2377 (18)	O4—H4B	0.872 (17)
C9—O3	1.2598 (19)	O5—H5A	0.834 (17)
C10—N3	1.3205 (19)	O5—H5B	0.879 (17)
			
C2—C1—C6	117.73 (17)	N3—C10—C11	123.54 (13)
C2—C1—C7	124.01 (14)	N2—C10—C11	117.56 (13)
C6—C1—C7	118.25 (15)	C12—C11—C10	120.83 (13)
C1—C2—C3	120.33 (18)	C12—C11—H11	119.6
C1—C2—H2	119.8	C10—C11—H11	119.6
C3—C2—H2	119.8	C11—C12—C13	119.33 (14)
C4—C3—C2	120.5 (2)	C11—C12—C15	120.87 (15)
C4—C3—H3	119.8	C13—C12—C15	119.79 (16)
C2—C3—H3	119.8	C14—C13—C12	118.45 (16)
C5—C4—C3	119.9 (2)	C14—C13—H13	120.8
C5—C4—H4	120.0	C12—C13—H13	120.8
C3—C4—H4	120.0	N2—C14—C13	121.56 (15)
C4—C5—C6	120.2 (2)	N2—C14—H14	119.2
C4—C5—H5	119.9	C13—C14—H14	119.2
C6—C5—H5	119.9	C12—C15—H15A	109.5
C5—C6—C1	121.4 (2)	C12—C15—H15B	109.5
C5—C6—H6	119.3	H15A—C15—H15B	109.5
C1—C6—H6	119.3	C12—C15—H15C	109.5
O1—C7—N1	121.22 (17)	H15A—C15—H15C	109.5
O1—C7—C1	121.09 (16)	H15B—C15—H15C	109.5
N1—C7—C1	117.66 (13)	C7—N1—C8	121.76 (13)
N1—C8—C9	115.75 (12)	C7—N1—H1	118.4 (13)
N1—C8—H8A	108.3	C8—N1—H1	119.7 (13)
C9—C8—H8A	108.3	C10—N2—C14	122.22 (13)
N1—C8—H8B	108.3	C10—N2—H2A	116.2 (11)
C9—C8—H8B	108.3	C14—N2—H2A	121.6 (11)
H8A—C8—H8B	107.4	C10—N3—H3A	121.3 (13)
O2—C9—O3	125.66 (13)	C10—N3—H3B	119.6 (13)
O2—C9—C8	120.40 (13)	H3A—N3—H3B	117.8 (19)
O3—C9—C8	113.94 (13)	H4A—O4—H4B	102 (2)
N3—C10—N2	118.90 (13)	H5A—O5—H5B	101 (3)
			
C6—C1—C2—C3	0.9 (3)	N3—C10—C11—C12	−179.07 (14)
C7—C1—C2—C3	179.70 (18)	N2—C10—C11—C12	1.2 (2)
C1—C2—C3—C4	−0.6 (3)	C10—C11—C12—C13	0.4 (2)
C2—C3—C4—C5	−0.3 (3)	C10—C11—C12—C15	−179.38 (15)
C3—C4—C5—C6	0.7 (3)	C11—C12—C13—C14	−1.5 (2)
C4—C5—C6—C1	−0.4 (3)	C15—C12—C13—C14	178.29 (17)
C2—C1—C6—C5	−0.5 (3)	C12—C13—C14—N2	1.0 (3)
C7—C1—C6—C5	−179.33 (17)	O1—C7—N1—C8	2.2 (2)
C2—C1—C7—O1	161.68 (17)	C1—C7—N1—C8	−175.96 (12)
C6—C1—C7—O1	−19.5 (2)	C9—C8—N1—C7	−87.11 (18)
C2—C1—C7—N1	−20.2 (2)	N3—C10—N2—C14	178.47 (15)
C6—C1—C7—N1	158.64 (15)	C11—C10—N2—C14	−1.8 (2)
N1—C8—C9—O2	−6.4 (2)	C13—C14—N2—C10	0.7 (3)
N1—C8—C9—O3	174.15 (14)		

### 2-Amino-4-methylpyridin-1-ium 2-(phenylformamido)acetate dihydrate Hydrogen-bond geometry (Å, º)

**Table:**

*D*—H···*A*	*D*—H	H···*A*	*D*···*A*	*D*—H···*A*
O5—H5*A*···O4	0.83 (2)	2.00 (2)	2.8225 (18)	167 (3)
N1—H1···O4^i^	0.85 (1)	2.25 (2)	2.9992 (18)	148 (2)
N2—H2*A*···O3^ii^	0.90 (2)	1.78 (2)	2.6850 (17)	176 (2)
N3—H3*A*···O2^ii^	0.89 (2)	1.99 (2)	2.8751 (17)	175 (2)
N3—H3*B*···O5^iii^	0.90 (2)	1.94 (2)	2.8368 (18)	171 (2)
O4—H4*A*···O3^iv^	0.88 (2)	1.88 (2)	2.7392 (17)	166 (2)
O4—H4*B*···O1^v^	0.87 (2)	1.95 (2)	2.8173 (18)	173 (2)
O5—H5*B*···O2^i^	0.88 (2)	1.94 (2)	2.8061 (18)	170 (3)

Symmetry codes: (i) −*x*+1, −*y*+1, −*z*+1; (ii) −*x*+1/2, −*y*+1, *z*−1/2; (iii) −*x*+1/2, *y*−1/2, *z*; (iv) *x*+1/2, −*y*+3/2, −*z*+1; (v) −*x*+1, −*y*+2, −*z*+1.
